# Some Observations on School Hygiene
1Read at the meeting of the Society held on April 12th, 1911.


**Published:** 1911-06

**Authors:** J. O. Symes

**Affiliations:** Late Physician to Clifton College; Physician to the Bristol General Hospital


### SOME OBSERVATIONS ON SCHOOL HYGIENE.[1]

BY

J. O. SYMES, M.D., D.P.H.

*Late Physician to Clifton College; Physician to the Bristol General Hospital.*

THE few remarks I purpose making this evening are concerned with the health of children at public and private boarding and day schools, and are in no way applicable to elementary or State-supported schools. In one way the latter class of school has a great advantage, inasmuch as they are subject to periodic medical inspection either by the medical officers of the Local Government Board, or by the medical inspectors of the municipality or county council. There is to my mind equal need of a Government inspection of public and private schools, in order that the drainage, closet accommodation, air-space and dietary may be brought up to a very much higher standard than at present. The children of parents of the middle class should obtain the same care from the State as those whose education is paid for out of public funds.

[1] Read at the meeting of the Society held on April 12th, 1911.

The duties of a school medical officer fall naturally under two heads: firstly, the superintendence of the general hygiene of the school with the view of developing the best possible physical well-being of the pupils; and, secondly, the care of children suffering from ordinary ailments. School authorities attach the greater importance to the second of these duties, and very largely the school medical officer becomes a drudge for small boys and girls suffering from mild minor ailments. The reason for this is that our medical schools make no provision whatever for instruction in the general principles which should guide us in the preservation of health, and the public upon questions relating to food, sleep, exercise, physical culture and mental training look for guidance outside the medical profession, and this part of our work is gradually falling into the hands of irregular practitioners. A very striking instance of this is Sandow's recent appointment as Director of Physical Culture to the King. I am aware, of course, that our own University provides a course of so-called "Hygiene," but this is taken up seriously by but few students, and is occupied with matters which in the ordinary course of things are the care of the plumber, sanitary inspector and engineer. What is needed is a course of instruction in what, for want of a better term, has been styled mechano-therapeutics.

The chief duties of a school medical officer should be the supervision of the drainage and sanitary fittings of the buildings, the regulation of the ventilation and air-space in the class-rooms and dormitories, the arrangement of the dietary, and the hours of work, play and sleep. It should also be his duty to report at the end of every term upon the physical condition of each pupil in exactly the same way as the master reports upon his mental condition. This would entail the taking of careful anthropometric records, and such are already taken in some of the Public Schools.

It is the duty of the medical profession to educate the public to expect school authorities to put physical culture and mental training on an equal scientific basis.

At Clifton College I was able to make a new departure in the matter of physical training, viz. the introduction of a master whose duty it was to undertake the treatment of physical defects. Each boy on entry presents himself for medical examination, and as a result of that examination a report is sent to his master stating what games he may play and to what extent he is capable of taking part in cross-country runs. Should a boy present any physical defect he was referred for special treatment to the director of physical exercises. The majority of cases so referred were boys with stooping gait, narrow chests or poor respiratory movement. These were all treated in one class, whilst cases of lateral curvature, flat-foot, deformity of chest, old infantile paralysis, asthma, etc., were taken individually for exercises, or for massage, or for both. In girls' schools the custom of providing a mistress trained in physical exercises has existed for some years, but much of the benefit of her services is lost by neglecting the routine initial medical inspection. It is very important that a suitable man be selected for the post of director of physical exercises. He must be efficiently trained not only in gymnastics, but in anatomy and in the elements of medicine ; he should have a pleasing personality, and should have the same social status as the other masters. His work should not be entirely confined to the play hours, but should be regarded as part of the usual school curriculum. I may here state that our Grammar School has recently made a notable advance in providing compulsory physical drill for every boy every day in school hours. I have seen the very greatest advantage accrue to boys from the services of the director of physical exercises, and a great improvement in the physique of the school generally follows upon the adoption of open-air Swedish drill, and the gradual elimination of the older gymnasium routine. I believe that such work is essentially the work for a medical man, and that we ought to provide the modern medical student with facilities for acquiring the knowledge and practice of methods of physical culture. We have at hand men qualified to teach this subject, and a new University, such as our own, should incorporate it as a course of postgraduate study.

*Sleep.*—Most of the Public Schools have recently made an important hygienic improvement in lengthening the hours of sleep. Speaking generally, the juniors should be allowed at least eleven hours and the seniors not less than ten hours in bed. Whilst this relief has been afforded and the actual hours devoted to study have been reduced, there can be no doubt that the pressure put upon the modern schoolboy is greater at the present time than ever before. If a curve of the sickness rate is kept term by term it will be noticed that even in the absence of epidemic disease there is a steady rise after the first two weeks followed by a fall during the last fortnight, when the routine of games and work is somewhat relaxed. I show such curves here this evening. In addition to having compulsory cricket eight or ten times a week, or football three or four times a week, the modern boy has in many instances to do military drills for the territorial service, fagging, swimming baths (summer and winter), fives, racquets, carpentry, singing, music, field club rambles, and attendance on various societies devoted to the discussion of politics, science and other subjects. The lazy or dull boy shirks many of these responsibilities, the keen-spirited boy wears himself out in trying to fulfil them. There is a real danger of a boy getting into mischief if left too long unoccupied, but by multiplying the daily duties we not only sap a boy's nervous strength and physical vigour, but we lessen the chance of his developing upon natural and original lines. Girls at school are given far more leisure than boys, and this is one of the reasons why they keep in much better health, and why their nervous and physical condition is improving more rapidly. Once a boy leaves school and enters the University this strain is removed, for games and hours of work are optional; consequently we see a pale, thin schoolboy converted into a ruddy, well-covered young man by a couple of terms' residence at the 'Varsity.

*Food.*—Another reason why the physique of schoolboys is unsatisfactory is that the feeding is injudicious. Deficiency of food is practically unknown in good class schools, but the nature of the food and the hours of meals might well be improved. The substantial meal of the day is dinner, given about one o'clock. After four hours' incarceration in classrooms, and after a visit to the grub shop during break, many boys have a poor appetite for this meal, and even such boys as are hungry hesitate about taking a heavy meal, fearing that their wind may be spoiled for the long afternoon run or game of football. For this reason I have advocated a substantial tea meal about six o'clock, a meal which in addition to the bread and butter, cake and jam served at present shall also include some proteid element such as eggs, fish, fish-cakes, rissoles, brawn, etc. This cannot be done without raising school fees, but most parents would prefer to pay a few pounds extra rather than be called upon to supplement the school tea as at present by a regular system of hampers. These two points that I have touched on, viz. the better regulation of meals and the reduction of the number of hours under school work and games, appear to me to call most urgently for attention at the present time.

With regard to the second division of the medical officer's duties, namely the care of sick children, one cannot fail to be struck by the infrequency amongst Public School boys of those scourges of the poorer classes rheumatism and tuberculosis. Of the many thousands of boys and girls who have passed through my hands during the past twelve years I have not detected one suffering from phthisis, abdominal tuberculosis or tubercular arthritis, and it would be possible to count up on the fingers of one hand the cases of tubercular adenitis.

Of 921 boys who have been under my care, and of whose past medical history I have notes, only 23 (2.5 per cent.) have had any symptoms of articular rheumatism, endocarditis, chorea or other rheumatic manifestation. One could not wish for a more striking demonstration of the fact that both these diseases are almost entirely dependent upon faulty conditions of hygiene.

There are two diseases almost entirely confined to Public Schools namely epidemic contagious impetigo and tinea cruris. Impetigo, popularly known as "scrum-pox" or "footer-mouth," closely resembles ordinary impetigo, but is peculiar inasmuch as it is highly contagious and exceedingly rapid in its progress. Starting generally as a slight excoriation on or behind the ears or on the malar bones, series of vesicles form upon the face, these again form a dry, flattened scab around which is a reddened, spreading areola. At the time the disease forms a most unsightly disfiguration, and the stained patches of skin may persist for many weeks after recovery. Rarely does the disease extend to other parts of the body. The disease only appears in epidemic form during the football season, and I have only seen it in those playing Rugby. The early excoriations are caused either by packing in the scrum or are received when tackling ; these are infected by the staphylococcus albus and aureus, which assume gradually an increased virulence. Subsequent cases arise by direct contact, or by contact with infected jerseys, towels or sponges. The men in the scrum suffer most, as one would expect. In a small outbreak of thirty-two cases, twenty-five were forwards, one half-back, four three-quarters, and two backs. Widespread epidemics of this disease sometimes occur in Public Schools, and as each case takes from two to three weeks to recover it leads to serious dislocation of school work if the patients are isolated. At first I isolated cases of impetigo, but soon found that boys recovered much quicker if attending school, and that when it was understood that infected persons were not granted sick leave, but were stopped playing football and subjected to somewhat unpleasant regulations, the spread of the disease was immediately stopped, and in the last four years it has never returned in epidemic form. The regulations roughly are the disinfection of all football clothes and of towels, sponges and basins ; no towels to be used in common ; infected boys to sleep in separate dormitories and use special lavatories ; attention to be paid to the slightest abrasion of the skin of the face and hands. The most successful treatment I found to be frequent bathing with antiseptic lotion, and then smearing with an ointment of equal parts of hazeline cream and ung. hyd. ammon. For obstinate and rapidly spreading cases an autogenous vaccine would probably be the quickest remedy.

Tinea cruris (ringworm of the groin and fork) is also a disease of the football season, due to a special fungus akin to tinea megalosporon. As I have recently read and published a paper on this subject I do not purpose saying more ; but communications sent to me since the publication of the paper show that this condition is getting a widespread prevalence in Public Schools, the Universities, and the services. It is spread entirely by the use in common of football clothes, towels, sponges and baths.

The infectious diseases are the great bane of school life, and whilst ordinary care should be taken to avoid their introduction, something needs to be done to prevent the wholesale waste of time and energy over futile and unnecessary precautions. For instance, in the case of German measles (roseola), the disease is never serious, and the patient might perfectly well be back at work in a week. There are so many mild unrecognised cases that it is useless to exclude contacts from school, and personally I think the medical profession might agree that this is an ailment of so trivial a nature as to demand no precautions whatever. Then again the code of the Medical Officers of Schools Association insists on disinfection of the person and clothes of children who have suffered or who have been in contact with such diseases as roseola, measles, mumps and whooping cough. This is of course a farce, and a source of trouble and expense to the parents. It would be well if the profession would clearly state that such procedures are useless. In private practice no one insists upon disinfection of persons who have been in contact with these milder epidemic diseases, and few submit patients convalescing from them to any such procedure. It is surely time that school medical officers did away with the restrictions now in force, which spread the belief that infection in these diseases can be conveyed in articles of clothing. Another futile and expensive procedure which might well be modified is the strict isolation of cases of measles, and the long period of quarantine enforced upon contacts. Armstrong has shown that with all the precautions taken only 2 per cent. of Public School boys escape contracting this disease. Whilst one would not advocate that boys suffering from measles should be allowed to mix with the others, it would, in my opinion, be permissible to abolish altogether the existing rules relating to quarantine, prolonged isolation and disinfection. Under the present arrangement the school routine is frequently upset for the sake of 2 per cent. or less than 2 per cent. of the boys. In connection with measles I should like to draw attention to the value of Koplik's spots in enabling one to detect cases four and even five days before the appearance of the rash. Other prodromal symptoms are slight enlargement of the cervical glands and the appearance of an erythematous rash on the face, neck and trunk on or about the second day. The early detection of cases of measles, whilst it does not limit the spread of infection, yet permits of the patient being put to bed early, and thus prevents many of the complications. Isolated cases of infectious disease, the origin of which cannot be traced and which do not give rise to other cases, are frequent in big schools. I have repeatedly had isolated cases of scarlet fever, chicken-pox and mumps which although they have been in contact with scores of other children yet have infected none. With regard to mumps, I think it should be recognised that parotitis and orchitis of a non-infectious nature are both common in boys at the age of puberty, and I have also seen two cases of acute thyroiditis and mastitis. The parotitis differs from mumps in that it is less painful and is confined to one side. Two out of the four cases of which I have notes had had mumps previously.

Of *albuminuria* in boys I need say very little. It is exceedingly common and bears no relationship to nephritis. It generally occurs in boys who need more out-door life and extra feeding. Of *hæmoglobinuria* after prolonged exertion I have had two cases. Both cleared in a few hours, and no ill-effects followed. It is probably the result of hæmolysis set up by toxins generated by over-exertion.

The most dangerous of all school diseases at the present time is *influenza*. Occurring during the spring term, not only does it dislocate school work by the absence of large numbers of pupils, but it endangers the life of one or more by the accompanying pneumonia, and leaves long-standing illness by complications such as otitis or cardiac weakness. The distinctive type of each epidemic is well seen in schools. In one epidemic pulmonary symptoms are common, whilst in another nine-tenths of the cases will be gastric or simply febrile. In 1907 epistaxis was a common premonitory symptom, whilst in 1909 enlargement of the cervical glands predominated. I have never had any success in preventing or lessening the incidence of the disease by the routine administration of quinine, but I am confident that the susceptibility of the children could be very much lessened if they led a more rational outdoor life during the Christmas holidays, if at school preparation of lessons before breakfast were abandoned for the winter months, and if greater discrimination were exercised in matters of food and clothing. It is more particularly in the winter that the substantial evening meal is required.

Before concluding there are two matters which I should like to touch upon. The first is the large amount of functional and organic nerve disorder which one finds amongst school children at the present time. Slight cases of epilepsy, tics, cases of periodic depression, of hysteria, and of mental and moral aberration at puberty are of frequent occurrence. It is a mistake to send children of abnormal nervous type to a Public School. They should either be sent as day scholars or be placed in smaller schools, many of which by adopting open-air methods and restricted hours of work are peculiarly favourable to such cases. The other subject I wish to refer to is that of long distance running and over-athleticism. Long runs should, of course, only be indulged in by boys who are physically sound, and who are carefully graduated according to age, size and ability. This is done at Clifton College by a medical examination, and by the selection of the masters and senior boys. I believe that such gradation is efficiently carried out, but with even the greatest care a boy must sometimes run when out of condition or force the pace too much. In this event there results an acute dilatation of the heart with rapid feeble pulse and signs of syncope. This is of short duration, and in three or four days no trace of the trouble can be discovered. Long runs have the effect of raising the blood-pressure, at first accelerating and then slowing the pulse. Possibly if this be repeated over a number of years it may lead to permanent damage, but I have never seen any ill-effects, and am of opinion that the dangers of over-athleticism have been much exaggerated.

